# Efficacy of Melatonin as a Promising Intervention for Migraine Prevention: A Systematic Review of Randomized Control Trials

**DOI:** 10.7759/cureus.72559

**Published:** 2024-10-28

**Authors:** Bhavana Nelakuditi, Bindu Jyothi Dandamudi, Kathrina Antheia M Dimaano, Nensi Shah, Osamah AlQassab, Zainab Al-Sulaitti, Safeera Khan

**Affiliations:** 1 Internal Medicine, California Institute of Behavioral Neurosciences & Psychology, Fairfield, USA; 2 Obstetrics and Gynecology, California Institute of Behavioral Neurosciences & Psychology, Fairfield, USA

**Keywords:** agomelatine, amitriptyline, headache disorders, melatonin, melatonin agonist, migraine, migraine disorder, migraine headaches, migraine prophylaxis, oral melatonin

## Abstract

The availability and use of melatonin as an over-the-counter supplement have surged significantly in recent years due to the increased prevalence of sleep-wake disorders, notably in the post-COVID-19 era. While melatonin is known for managing insomnia, its applications extend beyond that. Its anti-inflammatory, antioxidant, and analgesic properties, along with increased usage, have garnered significant interest from researchers, particularly regarding its use in migraine prophylaxis and treatment. The aim of this systematic review is to evaluate the role of melatonin as prophylactic therapy for migraine, focusing on the efficacy and side effect profile of melatonin compared to standard therapy and placebo. Six databases were searched through June 2024, identifying 735 relevant articles. Only full-text randomized control trials involving humans, written or translated into English, were included in the study. Data were extracted, screened, sought for retrieval, and assessed for quality appraisal using the revised Cochrane risk-of-bias tool for randomized trials (RoB 2). A total of seven randomized control trials involving 1,283 participants who met the eligibility criteria and passed the quality appraisal have been included in the study. All seven trials included patients diagnosed with migraine who were treated with either melatonin or agomelatine and were compared to those treated with conventional prophylactic therapy or placebo. The findings of this review suggest that melatonin significantly reduces the frequency and severity of migraines, but its dose-dependent action and benefits remain debatable. Melatonin may also have a role in weight control, warranting additional research in this direction.

## Introduction and background

Migraine ranks among the most prevalent neurological causes of disability, with an approximate global prevalence of 12%-20% [1-3]. Migraine episodes present as recurrent headaches, typically unilateral and accompanied by symptoms like nausea, vomiting, phonophobia, and photophobia. When associated with vision changes, tinnitus, speech disturbances, paraesthesia, extremity weakness, or stroke-like symptoms that are transient and fully reversible, it is classified as migraine with aura [4].

Migraine episodes can interfere with day-to-day functioning and compromise quality of life, warranting the need for prophylactic therapy. As per current recommendations, prophylactic agents are prescribed for patients having more than three episodes or at least eight symptomatic days per month [5,6]. Prophylactic therapy is also considered in migraineurs who are either resistant or intolerable to acute treatment [5-8]. Divalproex, topiramate, metoprolol, propranolol, and timolol are proven to be effective first-line prophylactic medications. Other drugs like amitriptyline, venlafaxine, atenolol, and nadolol are used as second-line treatments [4]. Calcitonin gene-related peptide (CRGP) inhibitors are being extensively studied and have recently been approved as prophylactic treatment for resistant cases [9]. These agents are expensive and can cause a financial burden for those opting for them [10].

A concerning fact is that 38% of patients need prophylactic treatment, but only 13% use it. This non-compliance can be attributed to the side effects of currently used regimens. Surveys show that migraine sufferers are among the most discontented patients. This warrants the necessity for newer preventive medications with fewer side effects [11-13]. Melatonin could be one such agent with a minimal side effect profile, which is also affordable [2].

In this systematic review, we set out to examine melatonin's role in preventing and treating migraines. We are specifically interested in the effectiveness of melatonin in reducing the intensity and recurrence of migraine episodes when compared to conventional prophylactic drugs like amitriptyline or placebo. Additionally, we will be discussing its side effect profile and tolerance. We will identify gaps in the existing literature and highlight potential areas for future research on melatonin and its impact on this condition.

## Review

Methods

This systematic review was conducted following the 2020 guidelines for Preferred Reporting Items for Systematic Reviews and Meta-Analyses (PRISMA) [14].

Search Sources and Strategy

We searched databases like PubMed, PubMed Central (PMC), Cochrane Library, ResearchGate, and ScienceDirect to find relevant literature published through June 2024 for our research question. Combinations involving melatonin and migraine were used to search all the databases. In PubMed Medical Subject Headings (MeSH) we employed ("Melatonin"[MeSh]) AND "Migraine Disorders"[MeSh], ("Migraine Disorders"[MeSh]) AND "Melatonin/therapeutic use"[MeSh] strategies to identify pertinent publications. We used an advanced search strategy utilizing the keywords "Melatonin” AND “Migraine" in PMC and Cochrane Library. On the ScienceDirect platform, we used "Melatonin AND Migraine" as keywords in both the title and abstract fields.

Table 1 below shows the databases used and the identified number of papers from each database.

**Table 1:** Search strategies and the number of papers identified from each database MeSH: Medical Subject Headings

Databases	Search strategy	Number of articles
PubMed	Melatonin AND Migraine	162
PubMed MeSH database	("Melatonin"[Mesh]) AND "Migraine Disorders"[Mesh], ("Migraine Disorders"[Mesh]) AND "Melatonin/therapeutic use"[Mesh]	105
PubMed Central	(Melatonin [Body - Key Terms]) AND Migraine [Body - Key Terms]	315
ResearchGate	Melatonin AND Migraine Disorders	100
Cochrane Library	Melatonin AND Migraine	19
ScienceDirect	Title, abstract, keywords: Melatonin AND Migraine	34
Total		735

Inclusion and Exclusion Criteria

Only randomized control trials involving humans were included in this study. The following criteria were also used to include literature: full-text articles either in English or with full text translated into English, research papers involving both natural and synthetic melatonin analogs (agomelatine), and any studies comparing the effects of melatonin with current migraine medications. Articles for which the full text could not be retrieved despite contacting the authors were excluded. Articles focusing on the therapeutic role of melatonin in other types of headaches were also excluded.

Screening

After conducting a preliminary search that produced 735 articles, we copied them to EndNote (Clarivate, Philadelphia, PA) and moved them to a Microsoft Excel sheet (Microsoft Corporation, Redmond, WA) to remove duplicates. A preliminary screening was conducted from the remaining list by thoroughly reading the titles and abstracts. This step was used to filter out irrelevant articles. The shortlisted articles were subjected to further screening. We evaluated the full texts to determine their relevance to our research question and applied specific inclusion and exclusion criteria to the remaining articles. Articles that fulfilled both criteria were shortlisted.

Quality Assessment of the Studies

The shortlisted articles were assessed with quality assessment using the revised Cochrane risk-of-bias tool for randomized trials (RoB 2). Only studies that met the quality appraisal criteria were finalized.

Results

Study Identification and Selection

**Figure 1:**
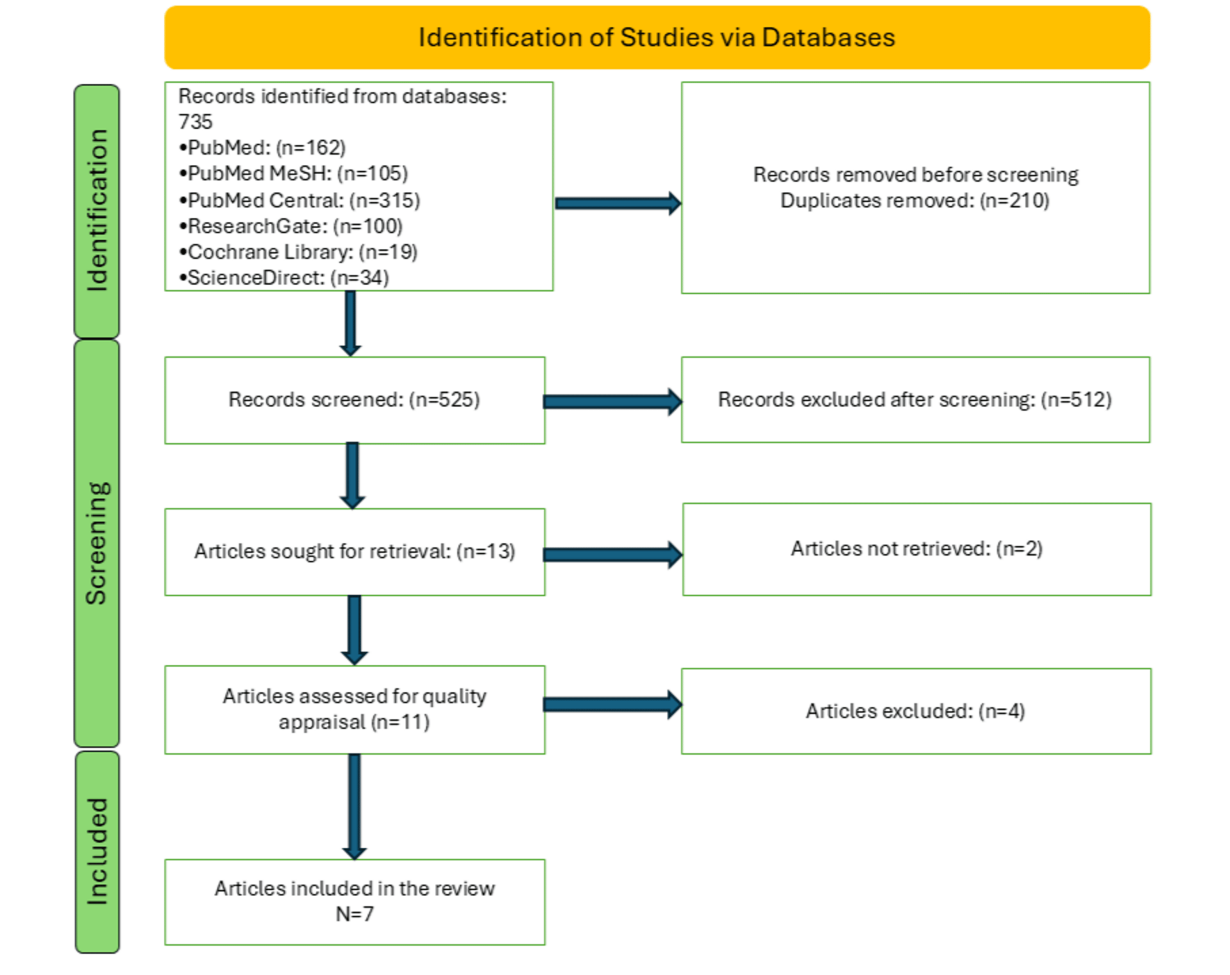
A PRISMA 2020 flowchart depicting the process of article selection MeSH: Medical Subject Headings; PRISMA: Preferred Reporting Items for Systematic Reviews and Meta-Analyses

We gathered 735 articles relevant to our topic from various databases; 210 duplicate articles were identified and removed before screening. A detailed screening process involving a thorough reading of titles and abstracts and application of inclusion and exclusion criteria resulted in 13 shortlisted papers. A total of seven randomized control trials with full-text articles that met the eligibility criteria and passed the quality check were included in the final review. The selection process is detailed below in Figure 1.

The articles were assessed using the relevant quality appraisal tool. Table 2 shows the results of the quality appraisal.

**Table 2:** Quality assessment of the studies using Cochrane bias assessment tool (RoB 2) + indicates a low risk of bias; - indicates a high risk of bias; ? indicates an unknown risk of bias

Study	Random sequence generation	Allocation concealment	Blinding of participants and personnel	Blinding of outcomes assessment	Incomplete outcome data	Selective reporting
Goncalves et al. [2]	+	+	+	+	?	+
Farzin et al. [15]	+	+	+	+	+	+
Mehramiri et al. [16]	+	+	+	+	-	+
Fallah et al. [17]	+	+	?	+	+	+
Nayeri et al. [18]	+	+	+	+	+	+
Alstadhaug et al. [19]	+	+	+	+	+	+
Gelfand et al. [20]	+	+	+	+	+	+

Outcomes Measured

The primary outcomes extracted from the final set of papers were melatonin use and the frequency and severity of migraine. The secondary outcomes include duration of headaches, comparison between melatonin and amitriptyline, tolerance to melatonin, and its side effects. Studies also included outcomes related to the effect of melatonin on migraine disability and sleep quality [15,16]

Study Characteristics

All seven articles finalized were randomized clinical trials and involved a total of 1,283 participants. All the studies included patients diagnosed with migraine who were treated with either melatonin or agomelatine. Two studies compared the effectiveness and tolerance of amitriptyline and melatonin in migraine patients [2,17]. Five studies included participants of all ages, whereas the remaining two studies exclusively studied the results in the adolescent population. Most of the finalized studies included all patients irrespective of the presence of aura, while two studies included patients with migraine without aura [15,18]. Women who were pregnant or lactating were excluded from four trials. Patients with chronic comorbid conditions like cardiac or liver disease were excluded from all the studies. One study exclusively incorporated individuals with migraine who received therapy for headaches but not maintenance therapy [18]. Two studies [2,19] excluded patients taking preventive therapy, whereas another [15] included those who did not take any preventive therapy for migraine. Table 3 presents a summary of the included studies.

**Table 3:** Summary of the included studies

Author	Type of study	Number of participants	Population characteristics	Intervention	Outcomes measured	Results and conclusion
Nayeri et al. [18]	Randomized control trial	50	Patients between the ages of 18 and 60 experiencing episodic migraines without aura	Administering 25 mg of agomelatine to patients in the intervention group for migraine management.	Agomelatine's potential to reduce migraine attacks' intensity and frequency.	Agomelatine is an alternate migraine preventative treatment option because of its favorable side-effect profile when compared to traditional migraine prophylactic drugs.
Farzin et al. [15]	Randomized control trial	400	Patients aged 18 to 60 with episodic migraines without aura, who had not received prior preventive treatment	The intervention group received 25 mg of agomelatine, while the control group was administered a vitamin B1 tablet as a placebo.	Impact of agomelatine on headache frequency and intensity, migraine-related disability (Migraine Disability Assessment (MIDAS)), and mean monthly migraine days (MMDs).	The number of headache days per month and mean monthly migraine days showed a significant difference between both the groups (control and intervention) three months following the intervention with p-values of 0.009 and 0.025 respectively. The number of episodes, mean monthly migraine days, migraine disability assessment (p<0.001), and headache severity (p<0.001) can all be reduced with the usage of agomelatine.
Fallah et al. [17]	Randomized control trial	80	Pediatric population aged five to 15 diagnosed with migraines and advised to undergo preventive treatment	Children were randomly assigned to receive either 1 mg/kg of amitriptyline or 0.3 mg/kg of melatonin for a duration of three months.	Change in frequency, intensity, duration, and disability scores of headaches. The frequency and severity of drug-related side effects, and the number of analgesics used were also assessed.	82.5% of the amitriptyline and 62%.5% of the melatonin groups showed a good response, and amitriptyline was significantly more effective (P=0.04). Both melatonin and amitriptyline are safe and effective for preventing migraines in children, however, amitriptyline is likely a better medication.
Mehramiri et al. [16]	Randomized control trial	81	Patients aged 20 to 57 diagnosed with migraines, with a male-to-female ratio of 25:60	The intervention group received a daily dose of 3 mg melatonin, while the control group was given a placebo of identical dosage, both alongside baseline treatment with propranolol 20 mg twice a day for two months.	Evaluations were conducted at baseline and in the first, second, third, and fourth months of follow-up to determine the frequency, duration, and severity of migraine attacks (as measured by the visual analog scale (VAS), the number of analgesics taken, drug-related complications, the score on the MIDAS, and the Pittsburgh Sleep Quality Index (PSQI).	The incidence and duration of migraine attacks were decreased by taking 3 mg of melatonin one hour before bedtime for two months. The amount of analgesics taken during the course of treatment was lower than with a placebo, and these beneficial effects persisted until the fourth month following treatment. Melatonin was found to be more effective than a placebo in reducing the frequency (p=0.032) and duration of migraine attacks (p=0.001). However, the severity of the symptoms remained the same in both groups (p=0.126).
Goncalves et al. [2]	Randomized control trial	438	Patients, both male and female, between the ages of 18 and 65 suffer from two to eight migraine attacks per month, either with or without an aura	Assigning participants to receive either 25 mg of amitriptyline, 3 mg of melatonin, or a placebo, with treatment carried out over a 12-week period.	The monthly migraine days between the baseline and final months were compared as the main outcome. Responder rate, the length and intensity of migraine attacks, and the use of analgesics were secondary objectives. Furthermore, the groups’ tolerance levels were also assessed.	In comparison to placebo, the melatonin 3 mg and amitriptyline 25 mg showed significantly higher efficiency (p<0.05) between the baseline and the last month of monitoring. For preventing migraines, melatonin (3 mg) works better than a placebo. Melatonin is superior to amitriptyline in the secondary endpoint (50% responder rate), although it is just as effective in the primary endpoint as 25 mg of amitriptyline. Compared to 25 mg of amitriptyline, it is more tolerable.
Gelfand et al. [20]	Randomized control trial	128	Adolescents with six to fourteen symptomatic days per month who are not receiving preventive treatment	For a duration of 12 weeks, 3 mg of melatonin was given nightly, while a placebo was administered to the control group.	Evaluate adverse events and use a home-trial methodology to determine the variations in headache results.	In the last four weeks, the melatonin group experienced fewer migraine days than the placebo group. This difference may have been caused by chance, but it did not reach statistical significance (mean difference, −1.3; 95% CI for difference, −5.1 to 2.6). An in-depth high-powered study is warranted. Home-based studies may be more advantageous for some patient populations and can hasten the development of neurological therapeutics.
Alstadhaug et al. [19]	Randomized control trial	106	Patients aged 18 to 65, with a male-to-female ratio of 3:20, experiencing two to seven migraine attacks per month.	Extended-release melatonin 2 mg was administered, one hour before bedtime, for a duration of eight weeks.	The frequency of migraine attacks (attack frequency (AF)) was the main outcome measured. The PSQI was used to measure sleep quality as a secondary objective.	The results of this study showed that 2 mg of prolonged-release melatonin taken an hour before bed does not prevent migraines any more effectively than a placebo, and as a result, this course of treatment cannot be recommended.

Discussion

Pathophysiology

Melatonin is a vital hormone that plays a crucial role that is essential for preserving the circadian rhythm and sleep-wake cycles. Hypothalamic dysfunction leading to low levels of melatonin in serum and urine samples of patients with migraine has been reported [21]. Owing to the relationship between sleep and headaches, both lack of sleep and excessive sleep are known to cause migraines [22]. Irregular production of melatonin is also linked to sleep disorders, which in turn are related to increased headaches. In addition, anxiety is a known trigger for primary headache disorders. Treatment of these disorders with melatonin indirectly has a positive effect on migraine [23,24].

The use of melatonin is recommended in migraine due to its anti-inflammatory, antioxidant, toxic free radical scavenging action, analgesic, and hypnotic properties. The negative effect on vasoactive substances, including CGRP, pro-inflammatory cytokine upregulation, and nitric oxide synthase activity [25], further adds to the beneficiary functions of melatonin. Inhibition of dopamine release, membrane stabilization, protection from glutamate-induced neurotoxicity, and neurovascular regulation further complement the therapeutic use of melatonin in migraine [26]. Due to its analgesic effect, melatonin should be used with caution in patients on opioids and opioid abusers [27]. Melatonin is known to cause a decrease in blood pressure and blood sugars. Therefore, dosage should be monitored when given to hypertensive [28] and diabetic patients [29]. Numerous scholarly articles have highlighted its antimigraine effects, noting that melatonin also protects the brain from toxic molecular damage. Its natural pain-relieving property, along with its ability to regulate circadian rhythms, makes melatonin a powerful preventive agent for reducing migraine attacks [30-32].

Synthetic Melatonin Analogues

New melatonin agonists have been developed in light of recent discoveries on melatonin receptors, with the goal of treating chronobiological conditions such as circadian rhythm sleep disruptions [33]. One such agonist is agomelatine, which is structurally like melatonin. Agomelatine is a potent MT1 and MT2 melatonin receptor agonist and has a selective antagonism against the 5-HT2c receptor. Agomelatine's dual action confers anti-inflammatory [34], vasodilatory [35], pain modulatory [36], and chronobiotic characteristics [37], hence supporting its involvement in the prevention of migraines.

Studies explored the possibility of oral agomelatine reducing the frequency and intensity of episodic migraine attacks in adults [15,18]. The effective dose of agomelatine for managing migraines is 25 mg per day [38]. The adverse effects are usually minor and bearable at this dosage [39]. However, one significant complication associated with agomelatine is dose-dependent and age-dependent hepatotoxicity [40]. In contrast, studies on administering melatonin to migraine patients have demonstrated that it is safe and typically has minimal or no side effects.

In clinical studies, the risk of hepatotoxicity is minimized by limiting the intervention to a short duration (three months), excluding patients over the age of 60 and individuals with a past diagnosis of liver disease. Furthermore, agomelatine is a reasonably safe alternative when used in conjunction with appropriate liver enzyme monitoring, as no significant drug interactions have been observed with it [41]. Given its favorable side-effect profile compared to conventional prophylactic medications for migraine, agomelatine is a viable alternative.

Melatonin vs. Amitriptyline

With a minimal side effect profile among the antidepressants, amitriptyline is most commonly used as a prophylactic therapy for migraine in children. However, it should not be used in children with electrocardiac abnormalities like arrhythmias and prolonged QT syndrome [42]. Amitriptyline could be particularly beneficial for treating depressed patients who also suffer from migraines. The adverse events of amitriptyline, such as sleepiness, can be reduced by prescribing a low dosage [43-45]

Goncalves et al. conducted a randomized clinical trial comparing the efficacy of melatonin and amitriptyline in migraine [2]. Results from this study showed that melatonin was as effective as amitriptyline in migraine prevention and better tolerated among the two. Melatonin also proved to be more effective than both the placebo and amitriptyline in achieving a greater than 50% reduction in migraine frequency for a higher percentage of patients. However, a study by Fallah et al. [17] concluded melatonin significantly reduced the frequency of headaches when compared to placebo (p = 0.009) but not when compared to amitriptyline (p = 0.19). A different study conducted in the USA discovered amitriptyline to be more effective than a placebo in reducing migraines for a maximum of eight weeks, but not for 12, 16, or 20 weeks [46].

Various other studies also showed a positive response rate for amitriptyline. Lewis et al. [47] showed a response rate of 89%, Hershey et al. [48] showed 84.2%, and a study by Kalita et al. [49] showed a 62% positive response in adults. In this study, 62.5% of children in the melatonin group experienced more than a 50% reduction in monthly headache frequency. [17]. In comparison, an Italian study [50] reported a 58% response rate and a Brazilian study [2] reported a 75% response rate. Factors such as drug dosage, race, age, sample size, and study design could account for these differences.

Interestingly, the side effect profile of amitriptyline when being used for migraine prophylaxis has been studied. A study in Dhaka [51], Bangladesh, showed minimal side effects when used for migraine prophylaxis. In this study, 22.5% of children experienced side effects such as daily sleepiness, constipation, and malaise in the amitriptyline group. Despite this, amitriptyline was generally well tolerated by children and didn’t have any serious adverse effects. Additional side effects such as mouth dryness, eye dryness, dizziness, and cardiac arrhythmia have also been documented in different studies [43].

When compared to amitriptyline, melatonin’s side effect profile is deemed better. Studies showed that children didn’t experience any serious life-threatening side effects with melatonin, and it was well tolerated [50-53]. However, daytime sleepiness was reported in a study in Sao Paulo, Brazil [2]. Other reported side effects of melatonin included worsening sleep patterns, agitation, behavioral changes, hyperactivity, seizures, nightmares, constipation, hypotension, and other sleep disorders [2,26,54,55].

Comparative Analysis of the Results Across the Studies

Farzin et al. [15] concluded that agomelatine is effective in the treatment of migraine in patients without aura (p = 0.009). Agomelatine was also deemed to be significant in reducing the frequency of migraine attacks in another study by Nayeri et al. [18], Mehramiri et al. [16], and Goncalves et al. [2] found that taking melatonin before bedtime significantly reduced the frequency and severity of migraines, as well as improved sleep quality (p<0.001 and p=0.009, respectively). Amitriptyline was found superior to melatonin in the treatment of migraine in one study, but melatonin was well tolerated (p = 0.04) [17]. A home-based study on adolescent migraines [20] showed a reduction in migraine frequency among adolescents who took melatonin daily but was not statistically significant (difference, −1.3; 95% CI for difference, −5.1 to 2.6).

Another study [19] indicated that 2 mg of prolonged-release melatonin did not improve migraine prevention compared to a placebo. Alstadhaug et al. concluded that melatonin did not offer significant benefits for migraine prevention (absolute risk reduction was 3%, 95% CI -15 to 21). Of note, most studies employed 3 mg of melatonin, but Alstadhaug and others used a 2 mg slow-release formulation [2,16]. The prolonged-release formula was chosen because two previous double-blind, placebo-controlled studies have shown a remarkable improvement in the quality of sleep and morning alertness with this dosage in patients over the age of 55 with insomnia [56,57].

Overall, variations in patient characteristics and study methodologies, including factors such as patient age, treatment regimens, and methods for measuring outcomes, might have contributed to differences in study results. When compared to a placebo, taking 3 mg of oral melatonin before bedtime dramatically reduced the incidence and intensity of migraine attacks, according to a meta-analysis by Tseng and associates (-1.71 days, 95% CI: -3.27 to -0.14). There were no adverse effects noted [58].

Scope in the Future

In studies by Nayeri et al. [18] and Farzin et al. [15], patients with migraine without aura were only included. The occurrence of ischemic stroke is significantly higher in patients with migraine with aura [59]. Hence, preventive measures for these patients should focus on lowering the risk of cerebrovascular consequences in addition to the intensity and frequency of migraine attacks [18]. Future studies should evaluate agomelatine's effectiveness as a preventive treatment for migraine with aura compared to other drugs by assessing its effects on the cerebrovascular system using objective indicators, such as microvasculature imaging.

Another interesting observation was that the melatonin group experienced weight loss, while the placebo and amitriptyline groups showed slight weight gain. This finding warrants special attention and opens opportunities for further research. The weight loss effect of melatonin may be related to its anorexigenic action, which regulates hypothalamic pro-opiomelanocortin (POMC) gene expression [60].

## Conclusions

Melatonin's therapeutic and preventative benefits for migraine disorders were the focus of this systematic review. Our study concluded that while melatonin may reduce migraine frequency in certain patients, more well-conducted trials are needed to confirm its overall efficacy. Additionally, the dose-dependent action and benefits of melatonin remain arguable. Oral agomelatine, a melatonin analog, has shown promising and well-tolerated results in migraine treatment. This study stands out for comparing various randomized control trials, highlighting melatonin's key role in migraine management. We analyzed studies comparing melatonin with both placebo and amitriptyline, demonstrating positive results in both treatment and prophylaxis of migraines. Given the current lack of effective medications for this common condition, our research emphasizes melatonin's potential as an affordable and well-tolerated treatment option. A limitation of our study is the exclusion of full-text articles that were not retrievable from databases or authors. Further research is needed to determine the optimal dosage for migraine treatment and to explore the side effect profile of melatonin. Additionally, agomelatine's promising results merit further investigation in future studies.
